# Defining and describing birth centres in the Netherlands - a component study of the Dutch Birth Centre Study

**DOI:** 10.1186/s12884-017-1375-8

**Published:** 2017-07-03

**Authors:** M.A.A. Hermus, I.C. Boesveld, M. Hitzert, A. Franx, J.P. de Graaf, E.A.P. Steegers, T.A. Wiegers, K.M. van der Pal-de Bruin

**Affiliations:** 1 Department of Child Health, TNO, PO Box 2215, 2301 CE Leiden, the Netherlands; 2 Department of Obstetrics, Leiden University Medical Center, PO Box 9600, 2300 RC Leiden, the Netherlands; 3 Midwifery Practice Trivia, Werkmansbeemd 2, 4907 EW Oosterhout, the Netherlands; 4 Jan van Es Institute, Netherlands Expert Centre Integrated Primary Care, Wisselweg 33, 1314 CB Almere, the Netherlands; 5 Department of Obstetrics and Gynaecology, Erasmus University Medical Centre, PO Box 2040, 3000 CA Rotterdam, the Netherlands; 6 Division Woman and Baby, University Medical Centre Utrecht, PO box 85500, 3508 GA Utrecht, the Netherlands; 7 NIVEL (Netherlands Institute for Health Services Research), PO Box 1568, 3500 BN Utrecht, the Netherlands; 8 Wijde Omloop 32, 4904 PP Oosterhout, the Netherlands

**Keywords:** Birth centres, Delivery rooms, the Netherlands, Midwifery, Definition, Midwifery unit, Midwife-led unit

## Abstract

**Background:**

During the last decade, a rapid increase of birth locations for low-risk births, other than conventional obstetric units, has been seen in the Netherlands. Internationally some of such locations are called birth centres. The varying international definitions for birth centres are not directly applicable for use within the Dutch obstetric system. A standard definition for a birth centre in the Netherlands is lacking. This study aimed to develop a definition of birth centres for use in the Netherlands, to identify these centres and to describe their characteristics.

**Methods:**

International definitions of birth centres were analysed to find common descriptions. In July 2013 the Dutch Birth Centre Questionnaire was sent to 46 selected Dutch birth locations that might qualify as birth centre. Questions included: location, reason for establishment, women served, philosophies, facilities that support physiological birth, hotel-facilities, management, environment and transfer procedures in case of referral. Birth centres were visited to confirm the findings from the Dutch Birth Centre Questionnaire and to measure distance and time in case of referral to obstetric care.

**Results:**

From all 46 birth locations the questionnaires were received. Based on this information a Dutch definition of a birth centre was constructed. This definition reads: “Birth centres are midwifery-managed locations that offer care to low risk women during labour and birth. They have a homelike environment and provide facilities to support physiological birth. Community midwives take primary professional responsibility for care. In case of referral the obstetric caregiver takes over the professional responsibility of care.” Of the 46 selected birth locations 23 fulfilled this definition. Three types of birth centres were distinguished based on their location in relation to the nearest obstetric unit: freestanding (*n* = 3), alongside (*n* = 14) and on-site (*n* = 6). Transfer in case of referral was necessary for all freestanding and alongside birth centres. Birth centres varied in their reason for establishment and their characteristics.

**Conclusions:**

Twenty-three Dutch birth centres were identified and divided into three different types based on location according to the situation in September 2013. Birth centres differed in their reason for establishment, facilities, philosophies, staffing and service delivery.

## Background

Throughout the world, birth centres are regarded as homelike settings where women with uncomplicated pregnancies can give birth with a midwife with the assistance of a maternity care assistant (MCA). When complications arise or when medicinal pain relief is requested, referral to a hospital obstetric unit takes place [1–5]. Birth centres differ from hospital obstetric units in management, staffing and the absence of medical obstetrical services as induction of labour, pharmacological pain relief, continuous foetal monitoring and instrumental birth. In general, birth centres focus on a model of care (e.g. the midwifery model) which ensures continuity of caregiver, a family-centred approach and informed client participation in choices related to the management of care [1, 6, 7]. In some countries they have been implemented as a response to counter the medicalization of childbirth by putting into practice the philosophy that in most cases childbirth is a physiological process [1, 8]. There are various nomenclatures for the birth centre concept based on their location in relation to hospital obstetric services: freestanding from a hospital (separate from a hospital, within a non-obstetric hospital, ‘stand-alone’) or attached to/within a hospital (alongside, co-located, in-hospital, integrated within or on the same campus) [1–3, 8–11]. Besides this distinction, differences are seen in their founding philosophies [1, 9].

Dutch women, considered at the start of labour to have low obstetric risk, can choose the place where they want to give birth: at home or out of home. Out of home birth can take place within a hospital setting or in a birth location outside of a hospital. The woman’s own community midwife is the responsible caregiver during labour and birth, regardless the location. She works autonomous and independent in a local midwifery practice. To work as a midwife in the Netherlands four years of education at the midwifery academy (Bachelor) have to be completed. After that, you are obliged to register in a nationwide register for health professionals [12]. Dutch midwives have not been trained or educated as nurses. During childbirth the community midwife is assisted by a maternity care assistant (a vocational education of three years). The maternity care assistant is employed by a maternity care assistance organization. A woman is referred to secondary care if risk factors arise during any time from the start of the pregnancy, until the postpartum period or if medicinal pain relief is requested during childbirth. Secondary care is provided under the responsibility of an obstetrician and clinical midwives or trainee obstetricians can be involved. This risk selection and role division between the professions is based on the List of Obstetric Indications, a document that designates the appropriate level of care for more than a hundred obstetrical conditions [13, 14].

During the last decade, a rapid increase in the number of out of home birth locations has been seen in the Netherlands. Several factors may be responsible for this sudden increase: women’s choice for home birth has decreased in recent years, leading to a higher demand for alternative birth locations that could not be provided by hospitals [15]. Besides that, birth centres are assumed to be a birth location that could provide more organizational efficiency by integration of perinatal care with better use of maternity care assistance [16, 17]. Thereby birth centres are seen as a safe alternative place of birth with fast access to an obstetric unit in case of referral [12]. Identification of these ‘birth centres’ is challenging as the term itself is used loosely: not all locations that call themselves birth centre in the Netherlands are places where women can actually give birth [13–16]. The term is also used for locations that house for example community midwifery practices, maternity care assistance organizations and ultrasound facilities.

The varying international definitions for birth centre are not directly applicable for use within the Dutch obstetric system where the place of birth is interrelated with the clear role division between primary and secondary obstetric care.

Between 2013 and 2016 the Dutch Birth Centre Study was carried out to evaluate birth centre care provision and its effects on perinatal outcomes, experiences of clients and caregivers and economic outcomes [17]. This evaluation was not possible without a consistent definition of birth centres for the Netherlands and information about their characteristics regarding location, available equipment and services and the model of care provided.

This study is part of the Dutch Birth Centre Study and aimed to develop a standard definition of birth centre for use in the Netherlands in order to identify all Dutch birth centres and to describe their characteristics.

## Methods

The methods used in the development of the birth centre definition were 1) the primary data collection, 2) a literature review and 3) a consensus process.

### Data collection tools

Three different data collection tools were used. The first one was a short digital survey to make a basic selection of potential birth centres in the Netherlands. The second one was the Dutch Birth Centre Questionnaire, used to get more information about the characteristics of these presumed birth centres and the third tool was the semi-structured interview for the confirmation and elucidation of earlier findings.

#### Short digital survey for potential birth centres

This tool was developed to obtain information about the place of birth options for low obstetric risk women in the Netherlands. It enquired about the existence of a) a homelike location for birth services for b) low risk women, that c) differed from the conventional hospital labour and birth setting. It was sent to the chair of every group of obstetricians associated with each of the 98 hospitals with maternity care in the Netherlands and to the chair of the local midwifery peer group in the vicinity of each of those hospitals.

#### Development of the Dutch Birth Centre Questionnaire (DBCQ)

A measurement tool for use in the Netherlands was developed based on an Australian questionnaire used to study birth centres (Laws, 2009). Permission was obtained for this survey tool that contains questions regarding issues as staffing, founding philosophies and physical characteristics of birth centres. Additional questions were added relating specifically to birth centre care provision in the Netherlands. These covered issues as initiators, reason for establishment, estimated number of births in 2013, need for transfer in case of urgent referrals and judicial status. The DBCQ consisted of 150 questions and was used to collect data from birthing locations that were presumed to be birth centres. In January 2014 all selected birth centres were asked to provide the number of actual births that took place at the birth centre in 2013.

#### Semi-structured interviews

Semi-structured interviews were designed to gather information from directing managers of those birth locations that qualified as presumed birth centre. Topics addressed included aspects of management and clinical leadership. During these interviews, information received from the DBCQ was confirmed and additional information was collected regarding time and distance from the birth centre to the hospital obstetric unit. Depending on the local situation, the distance from the birth centre to the obstetric unit was measured by counting steps or by kilometres on a navigation system. Time for transfer by bed or car was measured using a stopwatch during a simulated referral with transfer situation. All interviews were conducted by one researcher (IB).

### Development of a definition for birth centre in the Dutch context

In March 2013, international definitions of birth centres were searched in Pubmed and common elements within these definitions were identified. Using literature and data from the DBCQ, the characteristics of these elements were identified for the definition. A concept definition for birth centre was developed and discussed with the Dutch Birth Centre Study research group. Members of this group included 2 professors of obstetrics, 4 senior researchers and 3 PhD-students, two of whom were midwives (one practising).

In addition, the Dutch Birth Centre Study Advisory Committee discussed and adjusted the concept definition until consensus was reached [17]. After a final agreement from the project group, the definition was finalized.

### Identification of Birth Centres

Between April 2013 and June 2013, the locations that might qualify as a birth centre were collected in collaboration with The Royal Dutch Organisation of Midwives (KNOV), College of Perinatal Care (CPZ) and STBN (foundation for project management and innovation in natal care). A call was also posted in the popular LinkedIn Group “Dutch birth care in motion” to obtain information about other potential birth centres.

The Short Digital Survey was sent to midwives and obstetricians working in the vicinity of the identified potential birth centres. If they responded positively for all three questions, the location was presumed to be a birth centre. This resulted in a list of presumed birth centres for the study.

Representatives from each presumed birth centre were contacted by telephone, informed about the study and asked to participate. The local manager of each birth location was the primary person asked to answer the DBCQ. If the local manager was not available, the Chair of the Board or a midwife associated with the birth location was asked to respond on behalf of the birth centre. In July 2013, the DBCQ was sent by email to all presumed birth centres. Non-responders were contacted again in August 2013. All answers to the open-ended questions were analysed by two researchers (MHe and IB) and categorized after consensus was reached.

The semi-structured interviews with managers of the presumed birth centres were conducted by one researcher (IB) between January 2014 and April 2015. In May 2015 all birth centres were identified made in line with the Dutch definition of a birth centre and based on the information from September 2013.

### Analyses

Descriptive data analyses were conducted using the Statistical Package for Social Sciences (SPSS) version 22.0 (SPSS Inc., Chicago, IL, USA).

## Results

In total, 93 birth locations were identified as potential birth centres. After completion of the short digital survey, 47 birth locations were excluded because they were not homelike (*n* = 35), did not differ from the conventional labour ward on the obstetric unit (*n* = 27) or were not accessible as a birth location for low risk women who start labour under care of a community midwife (*n* = 8). More reasons for exclusion could be appropriate for one birth location. The remaining 46 locations were considered to be presumed birth centres and received the DBCQ. All questionnaires were returned of which 44 were fully completed. Two questionnaires were returned incomplete because the questions were not applicable for these two birth locations as being a presumed birth centre.

### Definition of a Dutch birth centre

Seven recurring elements were found after review of international birth centre definitions: 1) population to be served, 2) responsible professional for care provided, 3) environment, 4) philosophy, 5) location in relation to the nearest obstetric unit, 6) need for transfer in case of referral and 7) management structure (midwife/obstetrician). Using the information from the DBCQ (Table 1), characteristics were identified and formulated for the seven elements.

**Table 1:** Characteristics of included birth locations as presumed birth centres

Topic	Content	Characteristics	Included birth locations *n* = 46 (%)^a^
Philosophy	Commitment to physiological birth and facilities that contribute to the fulfilment of that philosophy	Facilities for discomfort and pain management which are allowed to be used in primary care (bath, shower, massage, nitrous oxide and/or TENS)	46 (100)
		Facilities to encourage spontaneous pushing in non-supine positions (birth chair, birthing ball)	42 (91)
		Assistance for community midwife during labour and birth by a maternity care assistant	42 (93)
		Providing one-to-one support	23 (51)
Environment	Homelike	Alterable lighting / homelike atmosphere	46 (100)
		No ‘medical’ equipment in sight	26 (57)
Responsibility for care	Community midwife	A Dutch community midwife is an independent medical professional who has full responsibility for providing care for healthy low risk women during pregnancy, childbirth and postpartum. The midwife conducts antenatal assessments, supports women giving birth at a place of their choice (at home, in a birth centre or in a hospital), and provides post-natal care up to six weeks postpartum. If medical assistance is required, the midwife will refer the women to a secondary caregiver (obstetrician or paediatrician). Community midwives in the Netherlands have a greater degree of autonomy in relation to the other medical professions than do midwives in most countries, but only as far as the low-risk population is concerned.	46 (100)
Population	Low risk women	Low risk women are women with a singleton pregnancy of a child in cephalic presentation who start labour spontaneously between 37 and 42 weeks and who do not have any medical or obstetric risk factors that are an indication for secondary care, such as formulated in the so-called List of Obstetric Indications [12]. They can choose where they would like to give birth (at home, in a hospital or in a birth centre).	46 (100)
	Medium risk women	Medium risk women are low risk women with a “medium risk” indication. Due to a specific reason they are advised to give birth in hospital but still under community midwife led care. The official medium risk indications according to the so-called List of Obstetric Indications are postpartum haemorrhage or retained placenta after a previous birth.	23 (50)
Management	Midwifery managed	In the organizational structure it is formally established that an independent community midwife is leading in care content and organization.	23 (50)
	Obstetric managed	In the organizational structure the obstetrician is leading in care content and organization.	23 (50)
Physical transfer in case of referral	Always needed	By wheelchair, bed, car or ambulance	10 (22)
	Always with exceptions	By wheelchair or bed but for some urgent reasons an exception is made and the secondary caregiver (obstetrician or paediatrician) will enter the room	13 (28)
	Not needed	The obstetrician enters the room	23 (50)
Location in relation to obstetric unit	Freestanding	Separate from the obstetric unit, in a different building than the hospital with an obstetric unit	3 (7)
	Alongside	Separate from the obstetric unit but in a hospital with an obstetric unit	17 (37)
	On-site	On the same ward as the obstetric unit	26 (57)

^a^ due to one missing value some percentages are calculated based on available data

All 46 presumed birth centres could be considered as locations to serve low risk women under the care of a community midwife at the onset of labour in a homelike environment. They all reported commitment to physiological birth and provided methods to deal with discomfort and pain during labour and birth that are considered standard care in Dutch primary care midwifery practice.

Management differed between being midwifery managed and obstetrical managed. To stay in line with international definitions the advisory committee of the Dutch Birth Centre study advised to include only locations that were midwifery managed as one of the conditions for the definition of a birth centre. Midwifery managed was defined as: “In the organizational structure it is formally established that an independent community midwife is actively and constructively involved in policy making and organisation of the content of care.” Due to the large variations in answers in the questionnaire and the interviews for this question, we created a list of conditions of which at least one had to be applicable to fulfil this item. These conditions were: the independent community midwife should be either 1) the owner of the birth location; 2) the floor manager of the birth location; 3) a member of the board of the birth location; 4) a member of the board of an integrated organization in which the birth location is a participant or 5) participating in a committee which is responsible for the local care content of the birth location.

**Fig. 1:**
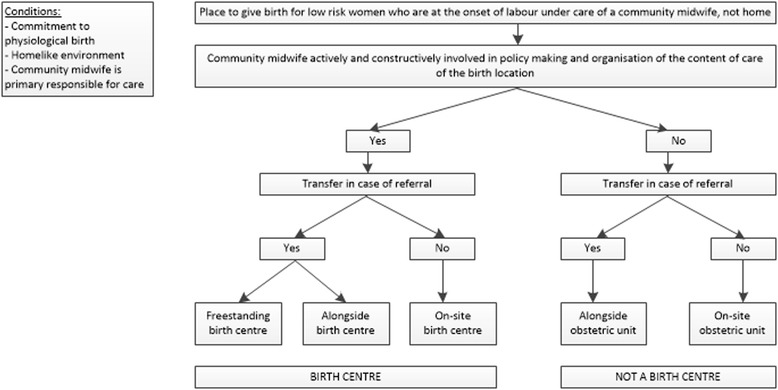
Flowchart for selection of type of birth location

The following definition of a birth centre was developed (Fig. 1):

- Birth centres are midwifery-managed locations that offer care to low risk women during labour and birth. They have a homelike environment and provide facilities to support physiological birth. Independent community midwives take primary professional responsibility for care. In case of referral the secondary caregiver (obstetrician or paediatrician) takes over the professional responsibility of care.

Three types of birth centres were identified based on location:

- A *freestanding birth centre* is located separate from a hospital with obstetric services. In case of referral the woman needs to be transferred to a hospital with obstetric services which will normally be by car or ambulance.
- An *alongside birth centre* is located in a hospital with obstetric services or on such a hospital’s grounds, but separate from the obstetric unit. In case of referral the woman needs to be transferred which will normally be by bed or wheelchair.
- An *on-site birth centre* is located within an obstetric unit of a hospital. In case of referral the woman does not need to be transferred: the secondary caregiver (obstetrician or paediatrician) will enter the birthing room.

### Selection of birth centres

**Fig. 2:**
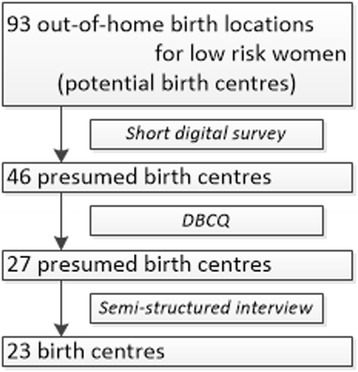
Flowchart for identification of Dutch birth centres. DBCQ: Dutch Birth Centre Questionnaire

Nineteen of the 46 presumed birth centres were excluded because they were not midwifery managed (see Fig. 2). Twenty seven presumed birth centres appeared to fit the definition based on the answers of the DBCQ. Their managers were interviewed and these locations were visited to confirm the fit of the definition and to obtain additional data. Another four birth centres were excluded because there was no involvement of the community midwife as defined in the definition. In total, 23 birth centres were identified in the Netherlands.

### Characteristics

#### Establishment

Most of the birth centres (*n* = 21) mentioned more than one reason for establishment. The most stated reasons were: the wish for a more homelike environment as opposed to conventional birthing rooms within the obstetric unit (74%), and the possibility to provide one-to-one support during early labour (57%). Competition and marketing were also mentioned as reasons; Ten birth centres (44%) were opened in order to compete with other hospitals offering a birth location for women with low obstetrical risk. Birth centres also mentioned logistics as a reason for establishment: in two regions (9%) the distance to a referral obstetric unit was perceived as being too large without the establishment of a strategically placed birth location for low obstetric risk women. Seven birth centres (30%) reported establishment because of a capacity problem in hospitals or in primary care services (shortages of birthing rooms at the conventional labour ward and shortages of midwives and/or maternity care assistants). More than three quarter (78%) of the birth centres reported that local community midwives were responsible for initiating the establishment of the birth centre.

#### Location

Table 2 shows that three birth centres were freestanding and two of them were located in a hospital without obstetric unit. In case of referral, the distance to the nearest hospital obstetric unit was between 3.7 and 30 km and took respectively 15 to 27 min by car or ambulance (from departure out of the birth centre to arrival at the obstetric unit).

**Table 2:** Characteristics of Dutch birth centres (September 2013)

	Freestanding birth centre *n* = 3	Alongside birth centre *n* = 14	On-site birth centre *n* = 6	Total *n* = 23 (%)
Length of operation (in years)				
< 2	1	7	4	12 (52)
2 to 6	1	5	1	7 (30)
6+	1	2	1	4 (17)
Location				
Not in a hospital	1			1 (4)
In a hospital without obstetric unit	2			2 (9)
Attached to a hospital with an obstetric unit		1		1 (4)
In a hospital on a different floor than the obstetric unit		5		5 (22)
In a hospital on the same floor but on a different ward than the obstetric unit		6		6 (27)
In a hospital on the same floor on the same ward as the obstetric unit		2	6	8 (35)
Number of women receiving intrapartum birth centre care in 2013 ^a^				
0–300	3	3	2	8 (35)
301–1000		8	2	10 (43)
1000+		2	1	3 (23)
No ‘medical’ equipment in sight	3	11	3	17 (74)
Birth chair	3	13	6	22 (96)
Medium risk-indications in birth centre			4	4 (17)
24/7 caregiver at birth centre	1	6	5	12 (52)
Moment of admittance at birth centre for women in labour				
As indicated by the woman	3	7	2	12 (52)
As indicated by the community midwife		7	4	11 (48)
Always	3	4		7 (30)
Always, with exceptions		10		10 (43)
Not needed			6	6 (27)
Birth assistance by a maternity care assistant (MCA)	3	14	6	23 (100)
One-to-one support by MCA	1	7	4	12 (52)
Possibility to stay over postpartum (without medical indication)	1	7	5	13 (57)
Change rooms postpartum for stay over		3	1	4 (17)
Hotel facilities in the birthing room				
Television	2	12	5	19 (83)
WiFi	2	14	4 (67)	20 (87)
Music-installation	3	10	3 (50)	16 (70)
Normal bed for partner	1	4	2 (33)	7 (30)
Coffee maker	3	12	3 (50)	18 (78)
Fridge	1	9	6	16 (70)
Microwave	2	10	1	13 (57)

^a^for two birth centres these data are not available because they started during 2013

Fourteen birth centres were located in a hospital but separate from its obstetric unit (alongside). In six of these birth centres referral with transfer to secondary care meant a move to another floor by elevator. Exceptions for transfer were locally described and included situations as shoulder dystocia (*n* = 9), resuscitation of the neonate (*n* = 8), postpartum haemorrhage (*n* = 4), (eclamptic) insult (*n* = 4), Apgar score below 7 after 5 min (*n* = 4), placental retention (*n* = 3), prolapse of the umbilical cord (*n* = 3) and foetal distress (*n* = 2). In those situations the secondary caregiver came to the birth centre in case of referral. In five of the 14 hospitals with an alongside birth centre there was also the possibility for low risk women to give birth under the care of their own community midwife on the conventional labour ward. The rooms on this ward were different in environment, staffing, service and facilities compared to the rooms in the birth centre. Transfer time from the alongside birth centre to the nearest obstetric unit varied between 10 s and 3.5 min.

Six birth centres were located within an obstetric unit (on-site). For low risk women who gave birth at an on-site birth centre transfer was not needed in case of referral because the obstetrician with the obstetric team entered the room. Besides the other conditions as noted in Fig. 1, they were distinctive from the conventional obstetric unit because of the active participation and responsibility of independent community midwives in the content of care and organization of this location. In case all beds in the obstetric unit were occupied the birthing rooms in the birth centre were used as obstetric birthing rooms as well. This was in contrast to the situation in freestanding and alongside birth centres.

#### Facilities to support physiological birth

All birth centres had a non-clinical homelike atmosphere. 74% of the birth centres have no medical equipment like a cardiotocography machine or a resuscitation bag and mask in sight. At the other birth centres this equipment in sight was minimalized by putting it not in a front position. All birth centres provided facilities to support pushing in a non-supine position (birthing stool, birthing ball), methods for discomfort and pain management that were allowed to be used in primary care (bath and shower) and one-to-one or one-to-two support by a maternity care assistant (MCA) as much as wanted and needed by the woman in labour and her partner.

#### Staffing

In all birth centres a MCA assisted the community midwife during labour, birth and postpartum. The MCA was part of the staffing of the birth centre in thirteen out of twenty three birth centres (57%). In twelve of these birth centres the MCA was 24/7 present. When not part of the staffing the MCA was on call for assistance during labour and came to the birth centre after a request by the community midwife. Midwives were not part of the staffing of the birth centre itself but were independent workers or part of the staffing of the larger organization that included the birth centre. They arrived at the birth centre only with a woman in labour or for postpartum care if applicable.

#### Family centred care

In thirteen birth centres (57%) it was possible for the woman to stay for up to 10 days postpartum. In four of these centres the woman stayed in the same room as where she gave birth; in the other she had to change rooms on the ward or in the building. In all except one of these thirteen birth centres it was possible for the partner to stay one or more nights as well if desired. During the postpartum stay, a maternity care assistant was available on the ward 24 h per day in every birth centre. Hotel-like facilities were present in all 23 birth centres.

#### Philosophies

Philosophies were ranked each from ‘not important’ to ‘very important’. The number of birth centres that ranked a philosophy as important or very important on the five point Likert scale are shown in Table 3 divided by type of birth centre. The philosophies ‘to provide a non-clinical homelike environment’ and ‘commitment to physiological birth’ were shared among all birth centres. These philosophies are part of the definition of a birth centre and the identification of birth centres was based on this definition. Two out of six of the on-site birth centres claimed that ‘minimal obstetric interventions’ was an important or very important philosophy for their birth centre. For the philosophy ‘minimal pharmacological pain management’ this was the case for three out of six of the on-site birth centres.

**Table 3:** Important or very important founding philosophies for birth centres

	Freestanding birth centre *n* = 3	Alongside birth centre *n* = 14	On-site birth centre *n* = 6	TOTAL *n* = 23 (%)
To provide a non-clinical homelike environment	3	14	6	23 (100)
To facilitate one-to-one/two support by MCA	3	14	5	22 (96)
Commitment to physiological birth	3	14	6	23 (100)
Encourage women’s rights and choices towards place to give birth	2	11	4	17 (74)
Encourage women’s rights and choices towards the way to give birth	2	13	4	19 (83)
Encourage family involvement	1	7	3	11 (48)
Minimal obstetric interventions	3	10	2	15 (65)
Minimal pharmacological pain management	3	10	3	16 (70)

#### Finance and legal entity

The establishment of the birth centres was financed in many different ways. In 55% the local hospital was involved, in 32% a maternity care assistance organization, in 23% an insurance company, in 23% STBN and in 14% the community midwives. For two locations this information was unknown by the person who filled out the questionnaire. In 61% the birth centre itself was an independent legal entity.

## Discussion

This study was undertaken to better understand the phenomenon ‘birth centre’ in the Netherlands. A standard definition for birth centre was developed, 23 birth centres were identified and their characteristics were described. Based on their location in relation to the nearest hospital obstetric unit, three different types of birth centres were seen: freestanding, alongside and on-site. Dutch birth centres differed in their reasons for establishment, services provided, founding philosophies, staffing and service delivery.

In the Netherlands, the term ‘birth centre’ has a broad scale of meanings, varying from midwifery practices to locations for preconception consults, which is confusing [13–16]. To have clarity about the term birth centre, we developed a definition for ‘birth centre’ for use in the Netherlands that is in line with international definitions, i.e. it is a place to give birth [1–5]. In general, there was not much discussion in the project group of the Dutch Birth Centre Study to describe the different options for the characteristics within the definition as provided by the answers of the DBCQ (as shown in Table 2) [17]. In the definition created for use in the English Birthplace study, the term ‘straightforward pregnancies’ was used to describe the group of woman who were eligible to give birth in a birth centre [5]. Although this was taken in consideration, it was decided that the term ‘low risk’ was a more appropriate term to use in the Dutch maternity system with its clear risk selection as written in the List of Obstetric Indications [18].

This is the first study in the Netherlands that looked into the classification and description of the characteristics of birth centres. With this classification, it will be possible to study the effects of birth centre care provision on many different aspects such as perinatal outcomes and client and healthcare provider satisfaction [19]. The interest in the evaluation of birth centre care in the Netherlands is shown by the enthusiastic participation with this sub-study by the professionals working in or with a birth centre. We identified all birth centres operating in September 2013 with some interviews held 1.5 year after filling out the DBCQ. Although it was specifically asked during these interviews to answer the questions as how the situation appeared at September 2013 some recall bias is not ruled out. It is important to acknowledge that as birth centres evolve quickly in number, location, organization and characteristics, current practice might already be different in some ways.

All Dutch birth centres claimed that it was important to be committed to a physiological way of birth. We found that at on-site birth centres medical equipment was more often in sight than in alongside or freestanding birth centres. In addition, as on-site birth centres are located on the obstetric unit, there is easy access to technology and medical interventions during labour and birth. Physicians working at the obstetric unit are trained to look for pathology, and maybe therefore more likely to intervene. Stark et al. found that the support of physiological labour and birth for low risk women when giving birth at the obstetric unit is more difficult than at another location different from the hospital obstetric unit [20]. Therefore, it might be more challenging to support physiological labour and birth at an on-site birth centre than at an alongside or freestanding birth centre.

Birth centres are homelike by having decorative changes like a specially designed bed and dim lighting and by providing hotel-like facilities. Facilities like a bath provide an option for non-pharmacological pain management that is associated with a significant reduction in risk of transfer and fewer interventions during labour [21, 22]. A birth environment that is calming and reduces stress supports physiological birth [23]. Although there is a wide variation in the interpretation of the element homelike among Dutch birth centres and the use of the facilities, birth centres could be a stimulating environment for midwives to give a stronger focus on physiological birth to enhance quality in Dutch maternity care. However, the creation of a culture that supports physiological birth involves more than the cosmetic appearance of the birth setting [20].

Worldwide there is discussion about safety and distance of travel time from a freestanding birth centre to a hospital with an obstetric unit in case of referral during birth [24–29]. Travel time differed from 5 to 60 min with a median of 15 min in Germany, to a median duration of 50 min in urgent situations in England [28, 29]. International studies showed that despite the time needed for a intrapartum transfer, planning to give birth in a freestanding birth centre significantly raised the likelihood of having a spontaneous, uncomplicated birth with good outcome for mother and infant [2, 25, 26, 29, 30–32]. In the Netherlands, referred low risk women with a travel time of at least 20 min had no higher risk of adverse outcomes [30]. In this study we found that some birth centres had been established in strategic locations to reduce travel time to secondary care. The maximum transfer time found was 27 min. Although international studies showed positive effects of travel time at freestanding birth centres and the travel time in the Netherlands is shorter, the effect of travel time for freestanding birth centres to obstetric units shall be studied in another part of the Dutch Birth Centre study [2, 17, 25, 26, 29, 30–32].

## Conclusions

It was possible to develop a comprehensive definition for a Dutch birth centre that is based on the common elements found in international definitions with context specific characteristics for the Netherlands. From the many locations calling themselves birth centres, it was possible to identify and select birth centres in line with our definition. This methodology has contributed to the ongoing research into the effects of birth centre care provision and could be valuable for future research in this area.

## Abbreviations

CPZ: College of Perinatal Care
DMCQ: Dutch Birth Centre Questionnaire
KNOV: The Royal Dutch Organisation of Midwives
MCA: Maternity Care Assistant
SPSS: Statistical Package for Social Sciences
STBN: Foundation for project management and innovation in natal care
